# The Role of Two-Pore Channels in Norepinephrine-Induced [Ca^2+^]_i_ Rise in Rat Aortic Smooth Muscle Cells and Aorta Contraction

**DOI:** 10.3390/cells8101144

**Published:** 2019-09-25

**Authors:** Sergei K. Trufanov, Elena Yu. Rybakova, Piotr P. Avdonin, Alexandra A. Tsitrina, Irina L. Zharkikh, Nikolay V. Goncharov, Richard O. Jenkins, Pavel V. Avdonin

**Affiliations:** 1 Koltsov Institute of Developmental Biology RAS, Moscow 119334, Russia; gad.91@inbox.ru (S.K.T.); alenka3107@mail.ru (E.Y.R.); ppavdonin@gmail.com (P.P.A.); sashulka.s@gmail.com (A.A.T.); 2 Institute of General Pathology and Pathophysiology RAMS, Moscow 125315, Russia; ilterekhina@gmail.com; 3 Sechenov Institute of Evolutionary Physiology and Biochemistry RAS, Saint Petersburg 194223, Russia; ngoncharov@gmail.com; 4 Research Institute of Hygiene, Occupational Pathology and Human Ecology, Leningrad Region 188663, Russia; 5 Leicester School of Allied Health Sciences, De Montfort University, The Gateway, Leicester LE1 9BH, UK; roj@dmu.ac.uk

**Keywords:** norepinephrine, calcium, two-pore channels, smooth muscle cells, NED 19, NAADP, siRNA, aorta

## Abstract

Second messenger nicotinic acid adenine dinucleotide phosphate (NAADP) triggers Ca^2+^ release via two-pore channels (TPCs) localized in endolysosomal vesicles. The aim of the present work is to evaluate the role of TPCs in the action of norepinephrine (NE), angiotensin II (AngII), vasopressin (AVP), and 5-hydroxytriptamine (5-HT) on free cytoplasmic calcium concentration ([Ca^2+^]_i_) in smooth muscle cells (SMCs) isolated from rat aorta and on aorta contraction. To address this issue, the NAADP structural analogue and inhibitor of TPCs, NED 19, was applied. We have demonstrated a high degree of colocalization of the fluorescent signals of *cis*-NED 19 and endolysosmal probe LysoTracker in SMCs. Both *cis*- or *trans*-NED 19 inhibited the rise of [Ca^2+^]_i_ in SMCs induced by 100 μM NE by 50–60%. IC_50_ for *cis*- and *trans*-NED 19 were 2.7 and 8.9 μM, respectively. The inhibition by NED 19 stereoisomers of the effects of AngII, AVP, and 5-HT was much weaker. Both forms of NED 19 caused relaxation of aortic rings preconstricted by NE, with relative potency of *cis*-NED 19 several times higher than that of *trans*-NED 19. Inhibition by *cis*-NED 19 of NE-induced contraction was maintained after intensive washing and slowly reversed within an hour of incubation. *Cis*- and *trans*-NED 19 did not cause decrease in the force of aorta contraction in response to Ang II and AVP, and only slightly relaxed aorta preconstricted by 5-HT and by KCl. Suppression of TPC1 in SMCs with siRNA caused a 40% decrease in [Ca^2+^]_i_ in response to NE, whereas siRNA against TPC2 did not change NE calcium signaling. These data suggest that TPC1 is involved in the NE-stimulated [Ca^2+^]_i_ rise in SMCs. Inhibition of TPC1 activity by NED 19 could be the reason for partial inhibition of aortic rings contraction in response to NE.

## 1. Introduction

The well-known mechanism of agonist-induced elevation of free cytoplasmic calcium concentration ([Ca^2+^]_i_) is its mobilization from endo/sarcoplasmic reticulum via InsP3- and ryanodine-sensitive channels. The latter is activated by cyclic ADP Ribose (cADPR) [1,2]. Somewhat later, NAADP was found to be another messenger that caused mobilization of calcium ions from intracellular depots [3]. This messenger released Ca^2+^ from a new calcium store: lysosomes and lysosome-related acidic compartments [4]. The Ca^2+^-mobilizing action of NAADP was shown for virtually all mammalian cell types examined [5]. The molecular target activated by NAADP is two-pore channels (TPCs), which are represented in human and mouse by two forms: TPC1 and TPC2 [6,7,8]. TPC2 is localized in lysosomes, and TPC1 is co-expressed with lysosome and endosome markers [8]. The function of these channels as a target of NAADP was described a decade after they were cloned [9]. TPCs are evolutionally ancient proteins that are present not only in animals, but also in plants, fungi and unicellular eukaryotes. In experiments with isolated lysosomes, it was demonstrated that both TPC1 and TPC2 were activated by PI-3,5-P2 while NAADP was without effect [10]. Jha et al. [11] have shown that both compounds stimulate TPC2.

The signaling pathways mediated by NAADP are involved in differentiation [12,13], histamine-activated von Willebrand factor exocytosis in human umbilical vein endothelial cells (HUVECs) [14], secretion [15,16], contraction of uterine smooth muscles in response to oxytocin [17] and detrusor muscle by carbachol [18], autophagy processes [19], etc. According to Brailoiu et al. [20], NAADP produces a dual effect in blood vessels: NO-dependent vasorelaxation in intact vessels and vasoconstriction in endothelium-denuded vessels. It was demonstrated that NAADP induces Ca^2+^ mobilization in cultured SMCs from pulmonary artery [21,22], and in these cells TPCs are involved in endothelin-1- and angiotensin II-induced [Ca^2+^]_i_ rise [22,23,24]. On isolated renal afferent arterioles, TPCs mediate a [Ca^2+^]_i_ rise in response to ET-1 or to norepinephrine [25]. However, information directly demonstrating the role of TPCs in vascular contraction in response to the agonists is limited—Zhang et al. [26] showed that bafilomycin A1 suppresses coronary artery constriction in response to ET-1. The aim of the present work is to clarify the role of TPCs in calcium signaling in rat aorta smooth muscle cells and in the regulation of rat aorta contraction by different agonists. To this end, we used *cis*- and *trans*-stereoisomers of NED 19, a specific antagonist of NAADP [27]. The relative efficacy of NED 19 stereoisomers was also assessed. Furthermore, in order to clarify the roles of TPC1 and TPC2 in receptor-dependent regulation of [Ca^2+^]_i_ in vascular SMCs we applied siRNA against these channels.

## 2. Materials and Methods

### 2.1. Reagents

*cis*-NED 19, *trans*-NED 19, U73122, and U73343 were acquired from Tocris (Bristol, UK); Fura2/AM, CalciumGreen/AM, and LysoTracker Red DND-99 from ThermoFischer Scientific (Waltham, MA, USA); Mitotracker Deep Red (M22426, Invitrogen), ER-tracker Red (E34250, Invitrogen), WGA Alexa Fluor 594 (Thermo Fisher, W11262), and antibodies anti-TPC1 (#ACC-071) and anti-TPC2 (#ACC-072) from Alomone Labs (Jerusalem, Israel); HRP-conjugated goat anti-rabbit IgG antibodies from BioRad (#170-6515); and the agonists of the receptors from SigmaAldrich (St. Louis, MO, USA).

### 2.2. Animals

Male Wistar rats between 250 and 300 g in weight were anesthetized with 25% urethane (4 mL/kg) and decapitated. The thoracic part of aorta was then removed. All manipulations with the animals were performed in accordance with the guide for the care and use of laboratory animals of the Bioethics Committee of the Koltsov Institute of Developmental Biology, and with the European Convention for the Protection of Vertebrate Animals used for Experimental and Other Scientific Purposes.

### 2.3. Registration of Aorta Contraction

Aorta was isolated from Wistar rats weighing 250–300 g, cleaned from connective tissue, and cut into rings with a width of around 2 mm. Experiments were performed on a 4-channel wire myograph (ADInstruments, Bella Vista, New South Wales, Australia) using the LabChart program for data acquisition and analysis. The rings were mounted on the holders in chambers filled with Krebs-Henseleit solution (in mM: NaCl—120; KCl—4.7; KH_2_PO_4_—1.1; NaHCO_3_—23.8; MgSO_4_—1.2; CaCl_2_—1.6; d-glucose—8) perfused with 95% O_2_/5% CO_2_ and extended with a force of 1 g at 37 °C. The contractility of the vessel rings and intactness of its endothelium were tested by adding 0.1 μM norepinephrine and then 10 μM carbachol. After washing, norepinephrine and carbachol were added again. Aortic rings showing relaxation by carbachol of at least 50% were used for the experiments. In the experiments with denuded aorta rings, the endothelium was removed by gently rubbing the intimal surface with a stainless-steel rod.

### 2.4. Cell Culture

Vascular SMCs were derived from aorta of male Wistar rats by an explant method as described previously [28] and cultured in Dulbecco’s Modified Eagle’s Medium (ThermoFisher Scientific, #11320074, Waltham, MA, USA) containing 10% fetal calf serum (ThermoFisher Scientific, #A31605).

Human umbilical vein endothelial cells (HUVECs) were isolated according to Xu et al. [29] with modifications [30]. The cells were grown in plastic dishes precoated with gelatin, using M199 medium with Earl’s salts and 20 mM HEPES (ThermoFisher Scientific, #11825015) containing 20% fetal calf serum (SigmaAldrich, F2442), 300 µg/ml endothelial growth supplement isolated from rabbit brain [31], 100 µg/mL heparin (SigmaAldrich, H3393), and 50 µg/ml gentamicin. We used the cells from early passages (2–3). For passaging the cells, accutase^®^ (SigmaAldrich, A6964) was applied.

### 2.5. Measurement of Free Cytoplasmic Calcium Concentration in SMCs and HUVECs

Measurement of [Ca^2+^]_i_ was performed on SMCs from 2 to 3 passages and on HUVECs from 2 to 4 passages grown in 48- or 96-well plates. The cells were loaded with 2 µM Fura-2/AM during one-hour incubation at 25 °C in a physiological salt solution (PSS) containing (in mM) NaCl (145), KCl (5), MgCl_2_ (1), CaCl_2_ (1), Hepes (5), and d-glucose (10) at pH 7.4. Pluronic F-127 (0.02%) was used to facilitate Fura-2/AM loading. Registration of the calcium signal was done in the cells incubated in PSS at 25 °C. The fluorescence was measured in parallel from three to six wells at excitation wavelengths of 340 and 380 nm and emission wavelength of 505 nm, using a Synergy 4 Microplate Reader (BioTek, Winooski, VT, USA). The increments in [Ca^2+^]_i_ are presented as ratios of the fluorescence at 340 and 380 nm.

### 2.6. Measurement of mRNA Coding TPC1 and TPC2 in SMCs

SMCs from passages 2 or 3 were used to analyze expression of the TPC1 and TPC2 genes. RNA from the cells was isolated using the Aurum™ Total RNA Mini Kit (Bio-Rad Laboratories, Hercules, CA, USA). The purity and concentration of isolated RNA were assessed by absorption at 260 and 280 nm using a NanoDrop 8000 spectrophotometer (Thermo Fisher Scientific, Waltham, MA, USA). The quality of the isolated RNA was evaluated by electrophoresis on a 1.2% agarose gel with ethidium bromide in TAE buffer (40 mM Tris, 20 mM acetic acid, and 1 mM EDTA). For reverse transcription, a High-Capacity RNA-to-cDNA Kit (Thermo Fisher Scientific, Waltham, MA, USA) was used. Real-time PCR was performed on a 7500 Real-Time PCR System (Thermo Fisher Scientific, USA) with reagent mixture qPCRmix-HS LowROX (Eurogen, Moscow, Russia). Beta-actin was used as a house-keeping gene for standardization. The following TaqMan probes and primers specific toward rat TPC1 and TPC2 were used.

TPC1-probe FAM-ACAGCCATCCAGCACGCATACCACC-BHQ1

TPC1-Sense CGACACCTTCAACGACATCG

TPC1-Antisense GCAGAGCAGCAACTTCATAAATATC

TPC2-probe FAM-TGCCCAGTTCATCTTCAGCCACCGC-BHQ1

TPC2-Sense CCGCCGAAGCCCCAGTAC

TPC2-Antisense AGCACCAGGAACACACAAATAGAC

### 2.7. Western Blot

SMCs grown in 48-well plates were lysed in RIPA-buffer (ThermoFisher Scientific, #89901) containing a protease inhibitor cocktail. The resulting lysate was centrifuged at 1000 rpm for 10 min, the supernatant was poured in aliquots and stored at −80 °C. The samples from the same lysate (20 μg of protein per well) were separated by 10% PAGE in Tris-Glycine buffer (25 mM TRIS-base, 250 mM Glycine, 0.1% SDS) and electroblotted onto PVDF membrane (BioRad Laboratories Inc., Hercules, CA, USA). After transfer, the membrane was blocked with 3% bovine serum albumin in PBS containing 0.02% Tween-20 for 2 h at room temperature, then cut into two parts: one of which was incubated with the antibodies against TPC1, and the other with antibodies against TPC2 in blocking buffer overnight at 4 °C. Then, both parts of the membrane were washed three times and incubated with horseradish peroxidase-conjugated goat anti-rabbit IgG antibodies (1:2500) for 1 h at room temperature. After incubation, the membrane was washed three times and developed using Li-Cor Western Sure UltraChemiluminescent Substrate and a C-Digit reader manufactured by LI-COR Biosciences (Lincoln, NE, USA). A Spectra™ Multicolor Broad Range Protein Ladder (#26634, ThermoFisher Scientific, Waltham, MA, USA) was used for the determination of protein molecular mass. The negative controls antigens were the peptides (C)RNLRQIFQSLPPFMD, corresponding to amino acid residues 221–235 of rat TPC1 (Accession Q9WTN5) and KKTLKSIRW(S)LPE(C), corresponding to amino acid residues 187–199 of mouse TPC2 with replacement of amino acid 192 with serine (S) (Accession Q8BWC0) that were used for the immunization.

### 2.8. Fluorescent Staining of TPC and Lysosomes in SMCs

The fluorescent probe *cis*-NED 19, specific for NAADP binding sites, was used for TPC staining in SMCs. The cells were incubated in DMEM FluoroBright medium (Thermo Fisher Scientific) with 20 μM *cis*-NED 19 and 75 nM of tracker (LysoTracker Red DND-99, Mitotracker Deep Red or ER-tracker Red), for 20 min at 37 °C in an atmosphere with 5% CO_2_ Cells, and then washed twice from unbound *cis*-NED 19 and tracers. Visualization was performed with a fluorescent microscope Zeiss AxioImager M1(Carl Zeiss, Oberkochen, Germany) with an objective Zeiss LCI Plan-NEOFLUAR 25×/0.8 DIC Imm Korr with water immersion. A Collibri diode illuminator was used as a source of exciting light. The following wavelengths were used: 380 nm for excitation of *cis*-NED 19 and 590 nm for LysoTracker, Mitotracker, or ER-tracker. The fluorescent signal was detected with appropriate filters (filter set 49 FT395 BP 445/50; filter set 62 HE BP585/35 LP615). From each sample, 20 images from 2 channels were taken. The resulting images were processed using the CellProfiler 3.0.0 software package containing a module for colocalization analysis.

For plasma membrane staining, SMCs were chilled for 20 min on ice in DMEM FluoroBright. The medium was then replaced with ice-cold, fresh medium, containing WGA Alexa Fluor 594 (Thermo Fisher, W11262) and 20 μM *cis*-NED 19. After 30 min of incubation, cells were washed with fresh cold DMEM FluoroBright media and imaged at +4 °C using a Linkam PE94 microscope controller stage (Linkam Scientific, UK). Imaging was performed by fluorescent microscope AxioImager M1 (Carl Zeiss, Germany) with an objective Zeiss LCI Plan-NEOFLUAR 25×/0.8 DIC Imm Korr with water immersion. A Collibri diode illuminator was used as a source of exciting light. The following wavelengths were used: 380 nm for excitation of cis-NED 19 and 590 nm for WGA Alexa Fluor 594. The fluorescent signal was detected with appropriate filters (filter set 49 FT395 BP 445/50, filter set 62 HE BP585/35 LP615).

To describe colocalization, we used the Rank Weighted Colocalization (RWC) coefficient [32]. The RWC coefficient for a pair of images R and G is measured as RWC1 = sum(Ri_coloc*Wi)/sum(Ri) and RWC2 = sum(Gi_coloc*Wi)/sum(Gi), where Wi is Weight defined as Wi = (Rmax − Di)/Rmax. Rmax is the maximum of Ranks among R and G based on the max intensity; Di = abs(Rank(Ri) − Rank(Gi)) (absolute difference in ranks between R and G); Ri_coloc = Ri when Gi > 0 and 0 otherwise; Gi_coloc = Gi when Ri > 0 and 0 otherwise.

### 2.9. siRNA Transfection

SMCs of passage 2 grown at 70–90% density in 48-well plates were transfected with siRNA of the following sequences, ACAACUGGGAGAUGAAUUA-dTdT (sense), UAAUUCAUCUCCCAGUUGU-dTdT (antisense) to suppress TPC1, and GGGUAAAUCCCGAGAACUU-dTdT (sense) and AAGUUCUCGGGAUUUACCC-dTdT (antisense) to suppress TPC2. As a negative control siRNA duplex (#1027310) from Qiagen was used. siRNA against TPC1 and TPC2 were prepared by DNA-Synthesis (Moscow, Russia, http://www.oligos.ru). siRNAs were mixed with Lipofectamine RNAiMAX (ThermoFisher Scientific, #13778150) in Opti-MEM medium (ThermoFisher Scientific, #51985026). The final concentration of each siRNA was 80 nM. The cells were incubated with siRNAs for 2 h, and then the media with siRNA was changed to growth medium for SMCs with serum. SMCs were used for [Ca^2+^]_i_ measurements three days after transfection.

### 2.10. Statistics

Statistical significance was calculated using the unpaired Student’s *t*-test and one-way or two-way ANOVA (MedCalc, Version 14.8.1, Ostend, Belgium); the values of EC_50_ were determined with GraphPad Prism 8 (Version 8.0.2, San Diego, CA, USA). Data are presented as means of independent measurements ± SEM. Independent measurements were performed on aortic rings isolated from different rats, on primary cultures of smooth muscle cells isolated from different rats or on endothelial cells isolated from different human umbilical veins. Within each independent measurement of [Ca^2+^]_i_ there were 4–6 parallel registrations, which were used to calculate average values for each point on the curves.

## 3. Results

### 3.1. TPC Expression and Localization in Rat Aorta SMCs

**Figure 1:**
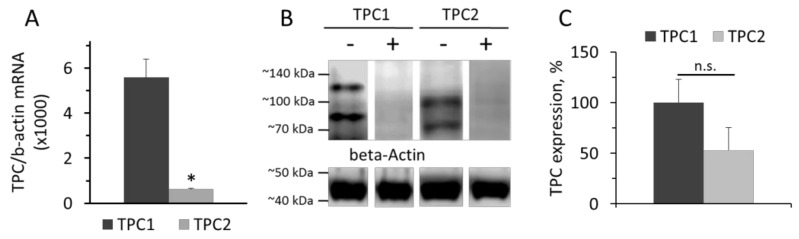
Levels of TPC1 and TPC2 mRNA relative to β-actin mRNA (**A**). Western-blot of TPC1 and TPC2 proteins (**B**) isolated from rat aorta SMCs and relative levels of TPC1 and TPC2 (**C**). The values panel A are the means of four measurements in different SMCs preparation + SEM (* *p* < 0.01). In panel B, the typical image of Western blots from one of three different cell lysates is presented. (+) Marks hybridization in the presence of blocking peptide. In panel C, n = 3 and n.s.—not significant.

Data on the levels of TPC1 and TPC2 mRNA in cultured rat aorta SMCs relative to β-actin mRNA are presented in the Figure 1. Levels of TPC1 and TPC2 mRNA were calculated from the ΔC_T_ value with β-actin as a standard. The levels of TPC1 and TPC2 mRNA in SMCs were 0.56±0.08% and 0.062+0.0047%, respectively, of the amount of β-actin mRNA (Figure 1A). The prevalence of TPC1 mRNA over TPC2 mRNA content was demonstrated earlier in denuded rat pulmonary artery and rat aorta [22]. However, at the protein level, the difference in the contents of TPC1 and TPC2 was not significant (Figure 1B,C). On Western blots, there are two protein bands stained with anti-TPC1 IgG, with molar masses around 80 and 125 kDa, and two bands stained with anti-TPC2 IgG with molar masses of approximately 70 and 100 kDa. Our results are similar to those reported previously by Churamani et al. [33]. Moreover, the authors showed that the determined molecular masses of TPCs vary over a wide range depending apparently on the degree of glycosylation. The presence of two bands on the Western is apparently because the sample contains glycosylated and deglycosylated forms of TPCs, as described earlier [22].

**Figure 2:**
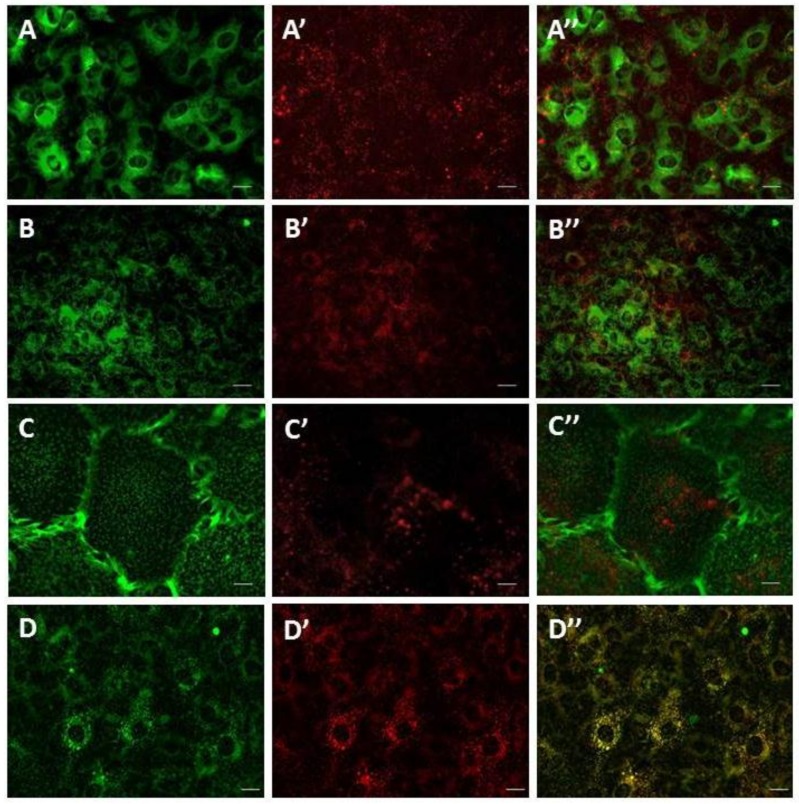
Live-cell imaging of *cis*-NED 19 localization in rat aorta smooth muscle cells. **A**—ER tracker; **A’**—*cis*-NED 19; **A’’**—merged. **B**—Mitotracker; **B’**—*cis*-NED 19; **B’’**—merged. **C**—WGA (plasma membrane traicer); **C’**—*cis*-NED 19; **C’’**—merged. **D**—Lysotracker; **D’**—*cis*-NED-19; **C’’**—merged. Scale—25 μm in **A**, **B**, and **D**, and 5 μm in **C**.

TPC1 and TPC2 expressed in SKBR3 cells are localized in the endolysosomal system [8]. To determine the subcellular localization of TPCs in SMCs, we used *cis*-NED 19, which is a ligand for NAADP binding sites. After 20 min incubation, *cis*-NED 19 was detected in the vesicular compartment of the cell. No fluorescence signal from *cis*-NED 19 was registered on endoplasmic reticulum, mitochondria, or plasma membrane (Figure 2A–C). The localization of *cis*-NED 19 fluorescence corresponded to the position of LysoTracker-stained lysosomes and lysosome-like vesicles (Figure 2D). To assess the degree of colocalization, two variants of colocalization analysis were applied (Table 1). In the first, vesicles containing the Lysotracker signal (colocalization inside the lysosome—lysosomes) as regions of interest (ROIs) were identified; in the second, vesicles containing the *cis*-NED 19 signal were identified as ROIs (NED-positive vesicles). To evaluate the degree of colocalization between *cis*-NED 19 and lysosomes, we used the Rank Weighted Colocalization (RWC) coefficient. The RWC coefficient considers not only the proportion of the colocalized pixels, but also their intensity. In the case of lysosome objects (lysosomes), both RWC *cis*-NED 19 and RWC Lysotracker coefficients were greater than 0.70. In the case of *cis*-NED 19 positive vesicles, these coefficients slightly decreased to 0.63. Difference between the two datasets can be explained by the existence of extralysosomal sites of *cis*-NED 19 accumulation. Nevertheless, these pairs of coefficients indicate a high degree of colocalization between the two signals in lysosomal compartment.

### 3.2. Study of the Effects of NED 19 on the Agonist-Induced [Ca^2+^]_i_ Elevation in SMCs

**Figure 3:**
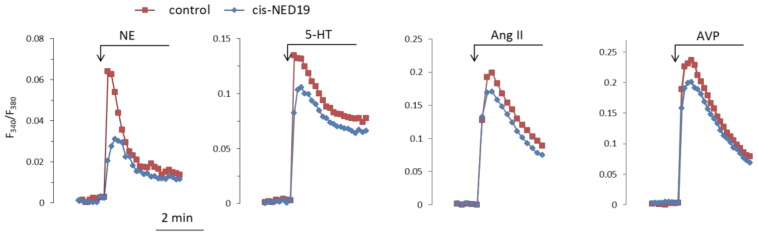
The kinetics of [Ca^2+^]_i_ changes in response to NE, 5-HT, Ang II, or AVP in rat aorta SMCs in the absence and presence of cis-NED 19. Concentration of *cis*-NED 19 was 5 μM in the case of NE application and 25 μM in other cases. Concentration of NE was 100 μM, 5-HT 10 μM, Ang II, and AVP 0.1 μM. The representative curves from one of four or more independent measurements are shown. Quantitative data are presented in Figure 4.

**Figure 4:**
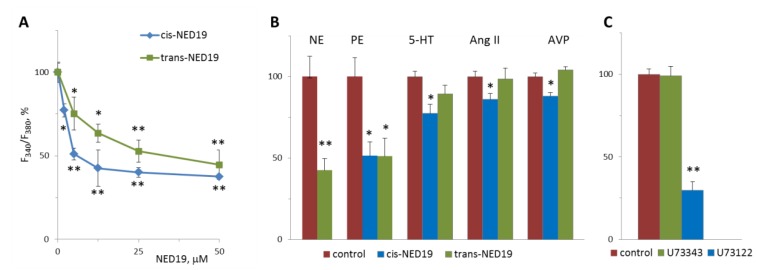
The effects of *cis*- and *trans*-NED 19 on the increase in [Ca^2+^]_i_ in SMCs in response to 100 μM NE (**A**), 10 μM NE, and 10 μM phenylephrine (PE), 5-HT, Ang II, and AVP, (**B**) and the effect of 1 μM U73122 and U73343 on the increase in [Ca^2+^]_i_ in SMCs in response to 100 μM NE (**C**). In panel B, the concentrations of the isomers of NED 19 were 50 μM with PE and 25 μM with 5-HT, Ang II, and AVP; the concentration of *trans*-NED 19 was 100 μM with NE. The concentrations of 5-HT, Ang II, and AVP were as in the legend to Figure 3. Each value is a mean of four to six measurements in different cell preparations + SEM. There is significant difference from control (* *p* < 0.05, ** *p* < 0.01).

Norepinephrine (NE), 5-hydroxytriptamine (5-HT), angiotensin II (Ang II), and Arg-vasopressin (AVP) induced a rapid elevation of [Ca^2+^]_i_ in cultured rat aorta SMCs (Figure 3). Ang II and AVP produced the greatest rises of [Ca^2+^]_I_; 5-HT causes an intermediate increase, whereas the effect of NE was much smaller. To evaluate the possible impact of TPCs on agonist-induced calcium signaling in SMCs, we used *cis*- and *trans*-NED 19. The cells were incubated with *cis*- or *trans*-NED 19 for 10 min before addition of the agonists. *cis*-NED 19 at 5 μΜ concentration suppressed maximal [Ca^2+^]_i_ increase in response to 100 μM NE by ~50% (Figure 3). There was slight decrease in [Ca^2+^]_i_ rises in response to 5-HT, Ang II, or AVP by 25 μΜ *cis*-NED 19. We compared the effects of *cis*- and *trans*-NED 19. As shown in Figure 4A, both stereoisomers at saturating concentrations of 25 and 50 μM, respectively, decrease the NE-induced [Ca^2+^]_i_ rise by nearly 60%. IC_50_ values were 2.7 μM (95% CI 1.0 to 7.2 μM) for *cis*-NED19 and 8.9 μM (95% CI 4.8 to 16.8 μM) for *trans*-NED19.

[Ca^2+^]_i_ elevation at 10 μM NE in the presence of 100 μM *trans*-NED 19 was 41.8 ± 6.3% of the control level (Figure 4B). A similar level of suppression of [Ca^2+^]_i_ elevation by 100 μM *trans*-NED 19 was elicited in response to 10 and 100 μM of NE, suggested an uncompetitive mechanism of inhibition. We investigated whether the action of NE was caused by the activation of alpha1-adrenergic receptors (α1-AR). An agonist of α1-AR, phenylephrine, induced [Ca^2+^]_i_ rise in SMCs, which was inhibited equally by both isomers of NED 19 at 50 μM concentration (Figure 4B). An agonist of β-AR, isoproterenol, did not cause elevation of [Ca^2+^]_i_ in SMCs (data not shown). The responses to 0.1 μM of 5-HT, Ang II or AVP were lowered by 25 μM *cis*-NED 19: by 22.7 ± 5.7, 13.9 ± 3.4 and 12.2 ± 2.3% respectively. There was no inhibition of the response to these agonists by 25 μM *trans*-NED 19. We assessed whether cleavage of phosphatidylinositol-4,5-bisphosphate is involved in the signaling pathway associated with NE-induced rise of [Ca^2+^]_i_ in the SMCs. In the presence of phospholipase C inhibitor U73122 at 1 μM concentration, the rise of [Ca^2+^]_i_ was decreased by 70.2 ± 6.4%.

The higher potency of *cis*-NED 19 as compared to *trans*-NED 19 in suppressing calcium responses to NE in SMCs was unexpected, as it was demonstrated earlier that the *trans*-isomer is approximately two orders of magnitude more potent than the *cis*-isomer as an inhibitor of NAADP-induced Ca^2+^ release in sea urchin egg homogenate [27]. To the best of our knowledge, the relative potency of *cis*- and *trans*-NED 19 as inhibitors of TPC in mammalian cells has not been studied previously.

**Figure 5:**
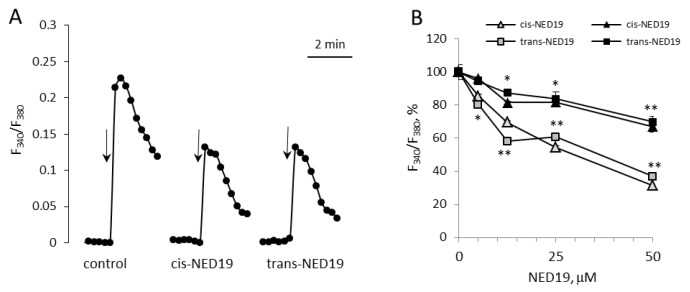
Decrease by *cis*- and *trans*-NED 19 of histamine-induced [Ca^2+^]_i_ elevation in HUVECs. (**A**) The kinetics of [Ca^2+^]_i_ rises in response to 1 μM histamine in the absence and presence of 25 μM *cis*- or *trans*-NED19; the arrows indicate histamine addition. (**B**) The effects of different concentrations of *cis*-NED 19 (triangles) and *trans*-NED 19 (squares) on [Ca^2+^]_i_ elevation in response to histamine at 1 μM (open triangles and squares) and 10 μM (black triangles and squares) concentrations. The values in panel B are the means of three to six independent measurements + SEM. There is a significant difference between control and experimental points (* *p* < 0.05, ** *p* < 0.01).

We compared the effects of *cis*- and *trans*-NED 19 on histamine-induced [Ca^2+^]_i_ rise in HUVECs, where the role of TPCs in calcium signaling has been demonstrated [14,34,35]. Preincubation of HUVECs for 10 min with either *cis*- or *trans*-NED 19 at 25 μM concentration resulted in a strong suppression of [Ca^2+^]_i_ rise in response to 1 μM histamine (Figure 5A). Both isomers of NED 19 exerted similar inhibitory effect. Concentration-dependence curves of the inhibition of [Ca^2+^]_i_ elevation in response to 1 μM histamine are almost identical for *cis*- and *trans*-NED 19 (Figure 5B). Inhibition of [Ca^2+^]_i_ rise in response to 10 μM histamine was weaker. However, the potencies of NED 19 stereoisomers were also equal. Thus, *cis*- and *trans*-NED 19 inhibited histamine-induced TPCs activation in HUVECs with the same efficiency. These data demonstrate the difference in the relative effectiveness of NED 19 stereoisomers in rat aorta SMCs and HUVECs towards Ca^2+^ signals induced by NE and histamine, respectively.

### 3.3. Inhibition of [Ca^2+^]_i_ Elevation in Response to NE by siRNA Against TPC1

**Figure 6:**
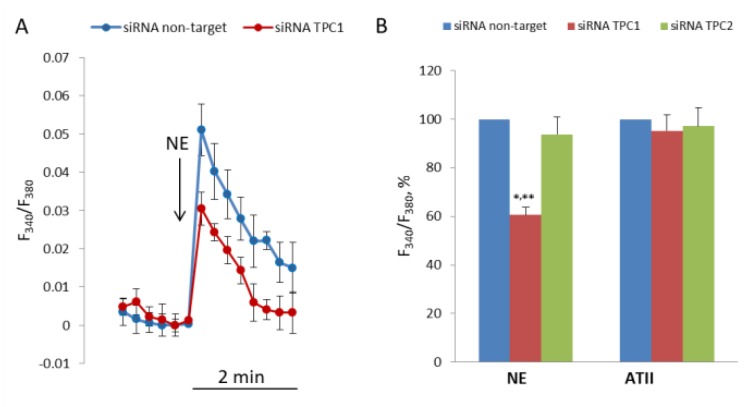
Decrease in [Ca^2+^]_i_ rise in response to NE in SMCs transfected with siRNA against TPC1. (**A**) Kinetics of [Ca^2+^]_i_ rise in SMCs transfected with siRNA against TPC1 and nontarget siRNA. The curves from one of four transfection experiments are presented. Each point at the curves is an average of six parallel measurements. (**B**) Calcium responses to NE (100 μM) and angiotensin II (0.1 μM) in SMCs transfected with nontarget siRNA, and siRNA against TPC1 and against TPC2. [Ca^2+^]_i_ rise in SMCs transfected with nontarget siRNA is taken as 100%. The mean values + SEM from four independent transfection experiments with different SMCs preparations are presented. There is significant difference between the responses of SMCs transfected with anti-TPC1 siRNA and with nontarget (** *p* < 0.01) or anti-TPC2 siRNA (* *p* < 0.05).

To evaluate the role of TPC1 and TPC2 in NE-induced elevation of [Ca^2+^]_i_ in SMCs, we transfected the cells with siRNA targeted against their mRNA. Transfection of SMCs with siRNA against TPC1 or with siRNA against TPC2 resulted in the decrease of TPC1 mRNA to 24.2% (21.9–26.6%, n = 3, *p* < 0.01) and TPC2 mRNA to 29.5% (27.1–32.2%, n = 3, *p* < 0.01), compared with SMCs transfected with nontarget siRNA. The levels of mRNA were measured 24 h after transfection, while the response to NE was studied in SMCs 72 h after transfection. As shown in Figure 6, after transfection with siRNA against TPC1 [Ca^2+^]_I_, elevation in response to NE was decreased by 39.3 ± 3.1% (*p* < 0.01). Conversely, suppression of TPC2 did not change NE-induced increase in [Ca^2+^]_i_ in SMCs. Knock down of TPC1 or TPC2 does not affect response to angiotensin II.

### 3.4. Study of NED 19 Effects on Rat Aorta Contraction in Response to NE, Arg-Vasopressin, Angiotensin II, and 5-HT

**Figure 7:**
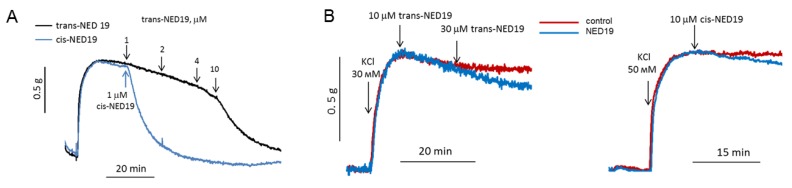
The influence of *cis*- and *trans*-NED19 on the contraction of aortic rings preconstricted by 0.1 μM NE (**A**) or KCl (**B**). (A) Each diagram is a representative of three or four independent experiments.

Based on the data that *cis*- and *trans*-NED 19 inhibit the NE-induced [Ca^2+^]_i_ rise in SMCs, we considered that they would suppress vasoconstriction in response to NE. The effects of NED 19 stereoisomers on the contraction force of rat aorta preconstricted with 0.1 μM NE are shown in Figure 7A. Both stereoisomers caused relaxation, with *cis*-NED 19 being the more potent, as it caused complete relaxation at 1 μM concentration, whereas *trans*-NED 19 exerted the same effect at much higher concentration of 10 μM. NE produces a sustained increase in the dihydropyridine-sensitive l-type Ca^2+^ current in vascular smooth muscle cells from rabbit ear artery [36], and the voltage-gated Ca-channels play a key role in alpha1A-adrenoceptor induced renal artery contraction [37]. We wondered whether vasorelaxation in response to NED 19 occurred due to inhibition of voltage-gated channels in SMCs. However, this possibility was excluded since aorta contraction in response to KCl was found to be only slightly inhibited by both *cis*- and *trans*-NED 19 (Figure 7B).

**Figure 8:**
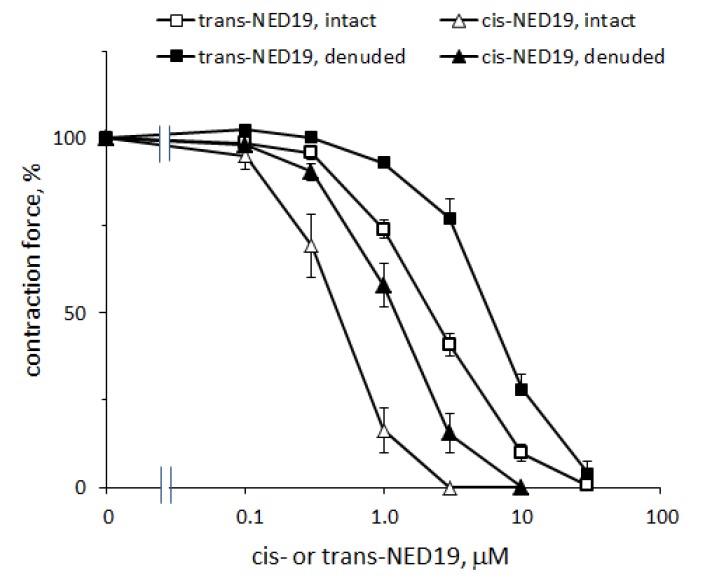
Concentration–response curves of aortic rings relaxation in response to cis- and trans-NED 19 with intact and denuded endothelium. The contraction force was measured 10 min after adding of each concentration of *cis*- or *trans*-NED 19. The data are the means + SEM from four independent experiments.

The concentration–response curves of the relaxation of aorta preconstricted by 0.1 μM NE caused by *cis*- and *trans*-NED 19 are shown in Figure 8. The IC_50_ for *cis*-NED 19 was 0.46 μM (95% CI 0.405–0.514 μM) and for *trans*-ED 19 EC_50_ was 2.33 μM (95% CI 2.01–2.72 μM). The relative efficiency of stereoisomers of NED 19 in suppressing the calcium signal elicited by NE (Figure 4A) was about the same. *Cis*- and *trans*-NED 19 also caused relaxation of denuded rat aorta rings preconstricted by NE. However, their effects occur at higher concentrations than in intact rings. The EC_50_ for *cis*-NED 19 increased to 1.25 μM (95% CI 1.14–1.36 μM) and for *trans*-NED 19 to 6.14 μM (95% CI 4.90–8.37 μM). This suggests that aorta relaxation might be partially endothelium-dependent. Another possible explanation is that in the presence of endothelium there is a tonic release of nitric oxide by endothelial cells [38], which potentiates the relaxing effect of *cis*- and *trans*-NED 19, while in denuded aorta there is no release of NO and the concentration–response curves shift to the right.

**Figure 9:**
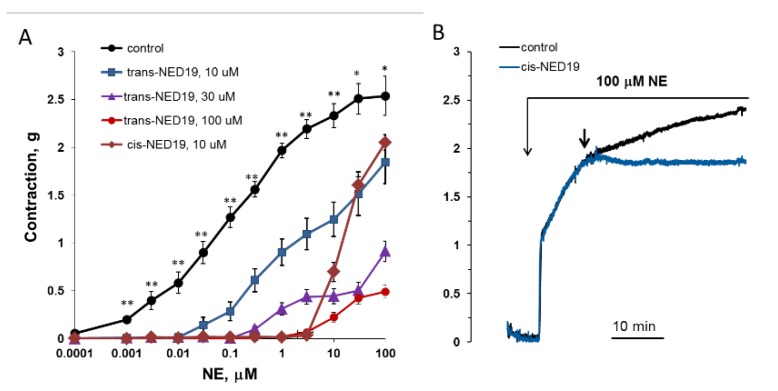
Contraction of rat aorta rings in response to increasing NE concentrations in the absence and presence of 10, 30, and 100 μM *trans*-NED 19 or 10 μM *cis*-NED 19 (**A**), and the influence of 30 μM *cis*-NED 19 on aorta preconstricted with 100 μM NE (**B**). Short arrow indicates addition of *cis*-NED 19 or vehicle. Each point is a mean of 8–12 independent measurements (8–12 rats) + SEM (* *p* < 0.05, ** *p* < 0.01 when compared with the contractions in the presence of *cis*- or *trans*-NED19). In panel B, the representative results from one of four experiments are presented.

The concentration–response curves of aorta contraction in response to NE in control conditions and in the presence of 10, 30, and 100 μM *trans*-NED 19 and 10 μM *cis*-NED 19 are shown on Figure 9A. *Trans*-NED 19 at 10 μM concentration shifted the concentration–response curve to the right by an order of magnitude. *Trans*-NED 19 at 30 and 100 μM caused further shift of the curve to the right. However, at these concentrations *trans*-NED 19 reduced the maximal force of contraction several-fold. The plateau phases in concentration–response contraction curves are clearly visible, suggesting an uncompetitive mechanism of inhibition. *Cis*-NED 19 inhibited contraction with higher potency than *trans*-NED 19. At 10 μM, *cis*-NED 19 completely suppressed contraction in response to NE at a concentration 3 μM or less. Conversely, 30 µM Cis-NED 19 only slightly inhibited 100 µM NE-elicited contraction (Figure 9B).

**Figure 10:**
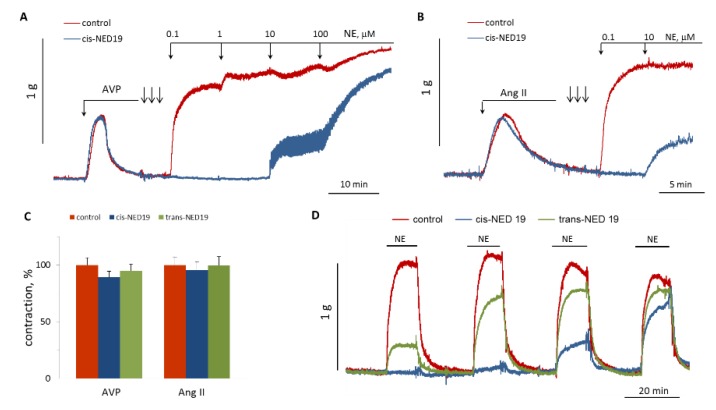
Comparison of NED 19 effects on the force of aortic rings contraction in response to Ang II, AVP and NE. (**A**,**B**) Lack of 30 μM *cis*-NED 19 effect on AVP- or Ang II-induced [Ca^2+^]_i_ rise and inhibition of NE calcium signaling. The rings were extensively washed from *cis*-NED 19, Ang II, or AVP (three arrows) before addition of increasing concentrations of NE. (**C**) Contraction of the rings in response to AVP or Ang II in the absence (100%) or presence of 25 μM *cis*-NED 19 or *trans*-NED 19. The values are the mean + SEM from four independent experiments. (**D**) The rings were incubated for 20 min with 10 μM *cis*-NED 19, *trans*-NED 19, or with vehicle before addition of 0.1 μM NE. Then, the rings were extensively washed and NE was added again. The procedure of washing and adding NE was repeated two more times. The typical results of one of three experiments are shown on each graph (**A**,**B**,**D**).

We studied the influence of *cis*- and *trans*-NED 19 on aorta contraction induced by Ang II or AVP. There were no effects on the contraction force in response to these agonists (Figure 10A–C). However, after extensive washing the rings from *cis*-NED 19 and AVP or Ang II (three arrows on Figure 10A,B), there was strong inhibition of the contraction induced by NE. We compared the reversibility of the effects of *cis*- and *trans*-NED 19 on aortic contraction caused by NE. As shown in Figure 10D, the suppression of NE-induced contraction by *cis*-NED 19 reversed very slowly; whereas, after washing *trans*-NED19 from the rings, the response to NE was restored almost completely after the first wash. These results correlate with data of Naylor et al. [27], according to which approximately half of Ned-19, which is a mixture of diastereomers, binds irreversibly to the NAADP receptor.

**Figure 11:**
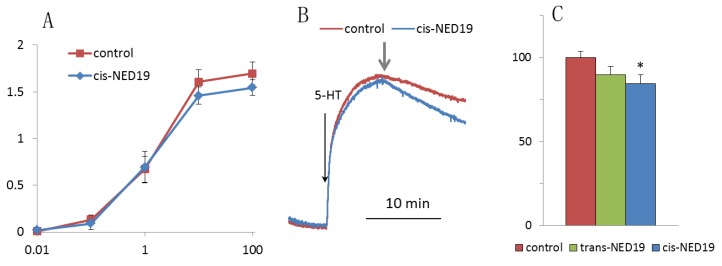
The effect of NED 19 on the force of aorta contraction in response to 5-HT. (**A**) Dependence of maximal contraction force on 5-HT concentration in the absence and presence of *cis*-NED 19. (**B**) Effect of *cis*-NED 19 on the force of aortic ring contraction in the tonic phase. Short arrow indicates addition of *cis*-NED 19 or vehicle. (**C**) Extent of inhibition of tonic contraction after 10 min incubation with NED 19 isomers is shown. Concentrations of *cis*- and *trans*-NED19 were 30 μM. Each value in panels (**A**) and (**C**) is a mean + SEM from the six independent experiments.

5-HT induced a rapid contraction of aortic rings, which was followed by slow relaxation. There was no significant effect of 30 μM *cis*-NED on the force of contraction induced by increasing concentrations of 5-HT (Figure 11A), whereas addition of *cis*-NED 19 to the rings preconstricted by 100 μM 5-HT resulted in a faster relaxation than in the control (Figure 11B,C). *Trans*-NED 19 did not produce a significant effect on the rate of aorta relaxation constricted by 5-HT.

### 3.5. Study of Bafilomycin A1 Effects on NE-Induced [Ca^2+^]_i_ Elevation in SMCs and on the Aortic Tone

**Figure 12:**
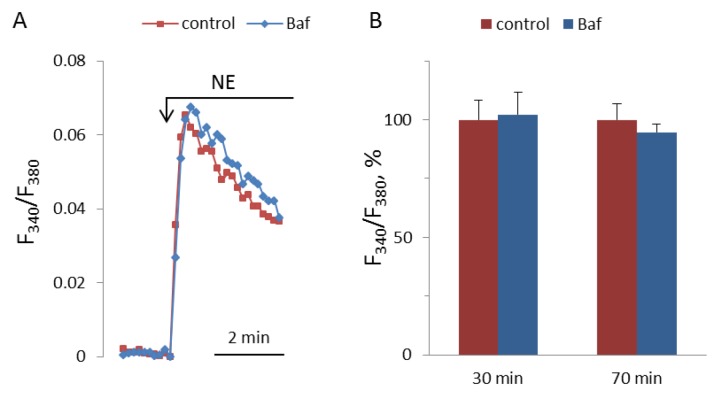
Kinetics of [Ca^2+^]_i_ rises in response to 100 μM NA in SMCs after 30 min preincubation with or without 0.5 μM bafilomycin A1 (**A**), and NA-induced [Ca^2+^]_i_ increments after 30 and 70 min preincubation with or without bafilomycin A1 (**B**). The rise of [Ca^2+^]_i_ in the absence of bafilomycin A1 is taken as 100%. Each value in panel B is a mean + SEM of six independent measurements.

NAADP-induced Ca^2+^ mobilization is disrupted by bafilomycin A1 in the pulmonary arterial wall [22], gastric smooth muscle cells [39], and hepatocytes [40]; whereas, in astrocytes [2] and T-lymphocytes [41], bafilomycin A1 does not inhibit the effect of NAADP on [Ca^2+^]_i_. In pulmonary artery SMCs, the depletion of lysosomal Ca^2+^ stores by bafilomycin A1 abolished Ca^2+^ signaling by ET-1 [23]. We incubated SMCs with bafylomycin A1 at 0.5 μM concentration for 30 and 70 min before addition of NE. In our experiments, bafilomycin A1 did not influence the [Ca^2+^]_i_ elevation induced by NE (Figure 12).

**Figure 13:**
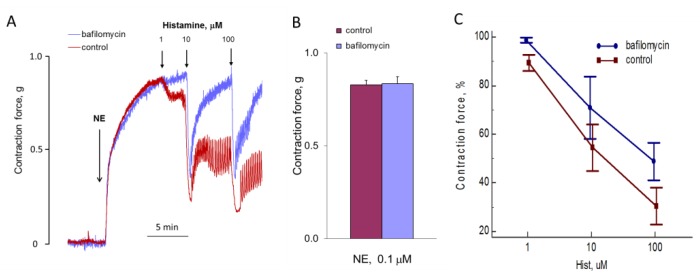
Influence of bafilomycin A1 on the effects of NE and histamine on aortic tone. (**A**) The kinetics of the contraction of the rings and relaxation in response to NE and histamine in the absence and presence of bafilomycin A1. Results of one of five typical experiments are presented. (**B**) The force of NE-induced contraction with or without bafilomycin A1. (**C**) The force of contraction (at points of maximal relaxation) in response to histamine of aortic rings incubated with or without 0.5 μM bafilomycin A1 during 1 h before adding of 0.1 μM NE. Values (B,C) are the means + SEM of five independent experiments. In panel C, there is a significant difference between control and bafilomycin A1 (*p* < 0.05).

We investigated the effect of bafylomycin A1 on aortic contraction under the action of NE (Figure 13). Incubation of the vessels for 1 h before the addition of NE had no effect on the speed and force of aortic ring contraction. Bafilomycin A1 can reduce the calcium signal of histamine in endothelial cells [14]. We tested whether bafilomycin A1 acts on histamine-induced aortic relaxation. In isolated aorta, bafilomycin A1 also inhibits the action of histamine, reducing the degree of relaxation.

## 4. Discussion

The purpose of this study was to determine the role of TPCs in the effects of vasoconstrictor agonists NE, 5-HT, Ang II and AVP on (1) the elevation of [Ca^2+^]_i_ in SMCs isolated from the rat aorta and (2) aorta contraction. In the first stage, we showed that TPC1 and TPC2 are expressed in the SMCs and that the amount of mRNA encoding TPC1 is an order of magnitude higher than that of TPC2. However, we did not reveal significant prevalence of TPC1 protein over TPC2 in SMCs from rat aorta. Note that Jiang et al. [22] reported similar proportion of TPC1 and TPC2 mRNA contents in denuded rat pulmonary artery and rat aorta. To evaluate the role of TPC1 and TPC2 in the receptor-dependent calcium signaling in SMCs, we applied siRNA targeted against the mRNA of these channels. We demonstrated that knock down of TPC1 decreased the response to NE, whereas the Ang II effect was not inhibited. Suppression of TPC2 did not affect NE calcium signaling. This is the first demonstration of the role of TPC1 in receptor-dependent [Ca^2+^]_i_ regulation in vascular SMCs.

NED 19, as a specific antagonist of NAADP [27], is widely used for study of the functional role of TPCs. In our experiments, to assess the role of TPCs in the realization of calcium signaling in SMCs, the *cis*- and *trans*-isomers of NED 19 were applied. We have demonstrated a high degree of colocalization of *cis*-NED 19 with lysosome and endosome marker LysoTracker. These results are consistent with previously published data on the endolysosomal localization of TPC1 and TPC2 in HEK293 cells [6] and in gastric SMCs [39]. We found that *cis*- and *trans*-NED 19 decreased NE-induced rise of [Ca^2+^]_i_ by nearly 60%. The degree of inhibition is higher than that obtained upon suppression of TPC1 with siRNA. This could be explained by incomplete inhibition of TPC1 by siRNA. We did not observe a decrease in NE-induced rise of [Ca^2+^]_i_ after transfection of SMCs with siRNA against TPC2. Our results are consistent with the data of Thai et al. [25], demonstrating suppression by NED 19 of NE-induced [Ca^2+^]_i_ elevation in isolated rat renal afferent arterioles. For 5-HT, Ang II, and AVP, the decrease in the maximal rise of [Ca^2+^]_i_ upon TPC blockade was much less pronounced, suggesting the lack or relatively small contribution of TPCs to calcium signaling from their receptors in rat aorta SMCs. The role of NAADP in calcium signals from receptors in different types of SMCs has been investigated previously. TPCs are involved in raising of [Ca^2+^]_i_ in response to endothelin-1 [22] and Ang II [24] in pulmonary artery SMCs, to carbachol in tracheal SMCs [42], and to oxytocin in rat uterine SMCs [17]. According to Govindan and Taylor [43], TPCs are not activated via P2Y purinergic receptors in aorta SMCs. In our experiments, in rat aortic SMCs, *cis*- and *trans*-NED 19 strongly inhibited the [Ca^2+^]_i_ rise in response to NE. However, in contrast to pulmonary artery SMCs there was a very weak inhibition of Ang II calcium signaling. These data suggest that the role of TPCs in receptor-dependent calcium signaling varies in different types of SMCs.

In our experiments, U73122, an inhibitor of phospholipase C that hydrolyzes phosphatidylinositol-4,5-bisphosphate, suppressed the increase in [Ca^2+^]_i_ by 70% in response to NE. This is consistent with data indicating that the main source of calcium ions releasing to the cytoplasm is the endoplasmic reticulum. It can be assumed that in rat aorta SMCs, calcium ions released from the endolysosomal system via TPCs in response to NE are likely to act as a trigger, enhancing mobilization of Ca^2+^ from the reticulum through the InsP_3_-sensitive channels. Such mechanism of NAADP-induced calcium elevation has been demonstrated in pulmonary artery SMCs [21,22] and in several other types of cells [44,45,46].

The action of NE is mediated by α1-AR, as phenylephrine also causes an increase in [Ca^2+^]_i_, which is inhibited by *cis*- and *trans*-NED 19. The increase of [Ca^2+^]_i_ through activation of α1-AR was demonstrated earlier in cultured canine femoral arterial SMCs [47] and in the SMCs of isolated rat mesenteric artery [48,49]. α1-ARs in SMCs are coupled to phospholipase C catalyzing the formation of InsP3, which in turn causes the release of Ca^2+^ from the reticulum [50,51]. In parallel, α1-AR agonists activate entry of calcium ions into the SMCs via TRPC6 triggered by diacylglycerol, which is another product of the phospholipase C reaction [52,53]. Our data demonstrate that in addition to these mechanisms TPC1 is also involved in the α1-adrenergic regulation of calcium ions in vascular SMCs.

The potency of *cis*-NED 19 in suppressing calcium signals in the SMCs was higher than that of *trans*-NED 19. In contrast, *trans*-NED 19 is approximately two orders of magnitude more potent than the *cis*-form as an inhibitor of NAADP-induced Ca^2+^ release in sea urchin egg homogenate [27]. We compared the effects of NED 19 stereoisomers in endothelial cells, where the involvement TPCs in the calcium signaling induced by histamine is well defined [14,34]. In HUVECs, *cis*- and *trans*-NED 19 reduced the rise of [Ca^2+^]_i_ caused by histamine with equal efficacy. To explain differences in effectiveness of *cis*- and *trans*-NED 19 in suppression responses to different agonists, it is hypothesized that it is due to the participation of different TPCs in calcium signaling from different receptors. There are data that indicate that TPC1 and TPC2 are differently regulated by NED 19 [54]. In this regard, it is worth noting that in endothelial cells TPC2 transmits a calcium signal from the VEGF receptors [35]. Concerning H1 histamine receptors, it has been shown that their activation of von Willebrand factor secretion decreased after simultaneous knockdown of TPC1 and TPC2 with siRNA [14].

We have demonstrated that both *cis*- and *trans*-NED 19 completely suppressed aortic contraction in response to low concentrations of NE. This suggests that TPCs are involved in rat aorta contraction caused by NE. With an increase in NE concentration, the inhibitory effect decreased, but even with high concentration of NE (100 μM) *cis*- and *trans*-NED 19 reduced the contraction force. This suggests that at high concentrations of NE the vasoconstriction is less dependent on TPCs.

It should be noted that the potency of cis-NED 19 in suppressing the contraction caused by NE was approximately 4–5 times higher than that of trans-NED 19; the same relative potency as found for calcium signaling suppression. The coincidence of the relative efficacy of *cis*- and *trans*-NED 19 in suppressing the calcium rise in SMCs and blood vessel contraction in response to NE, suggests that in both cases the same mechanism is involved. This in turn suggests a causal relationship between the rise of [Ca^2+^]_i_ in SMCs and aortic contraction induced by NE.

In our experiments, bafilomycin A1 did not suppress NE-induced elevation of [Ca^2+^]_i_ in SMCs and NE-induced contraction of rat aorta. A possible explanation is that in SMCs TPCs are localized in the vesicles, where the level of intraluminal Ca^2+^ can be maintained when the activity of V-type H^+^-ATPase is inhibited. According to our data, TPC1 is involved in NE-induced [Ca^2+^]_i_ elevation in SMCs. It has previously been demonstrated that TPC1 are localized predominantly in early endosomes with a less acidic intraluminal pH than in lysosomes, which express TPC2 [6,45]. In HUVECs, TPC2 mediate calcium signaling of vascular endothelial growth factor [35] and apparently are involved in H1 receptor signaling [14]. In HUVECs, bafilomycin A1 effectively inhibits [Ca^2+^]_i_ rise in response to histamine [14]. In our experiments, histamine-induced relaxation of rat aorta was partially attenuated by bafilomycin A1.

The lack of *cis*- and *trans*-NED 19 effects on aortic contraction force, in response to Ang II and AVP, shows that TPCs do not participate in the regulation of vascular tone by these agonists, or that their contribution is negligible. The slight suppression of the calcium response induced by them in SMCs under the action of *cis*-NED 19 does not appear to have a significant effect on the physiological response of the vessel. According to our data, TPCs are not involved in the rapid phase of aortic contraction in response to 5-HT. In the presence of *cis*-NED 19, the second longer phase of aortic contraction induced by 5-HT is attenuated.

Previously, the involvement of TPCs in contraction of uterine smooth muscles in response to oxytocin [17] and detrusor muscle by carbachol [18] was demonstrated. There is considerable amount of published data demonstrating TPCs involvement in receptor-dependent calcium signaling in vascular SMCs [21,22,23,24,55,56,57]. However, currently just fragmentary information is available on the role of TPCs in the regulation of vascular tone. Brailoiu et al. [20] demonstrated that NAADP-AM induced NO-dependent vasorelaxation in aorta with intact endothelium and vasoconstriction in endothelium-denuded vessels. Zhang et al. [26] show that bafilomycin A1 decreases bovine coronary artery constriction in response to ET-1. Our results provide evidence that TPCs are involved in vasoconstriction caused by NE. Experiments on SMCs with siRNA suggest involvement of TPC1 in this physiological reaction.

## Figures and Tables

**Table 1:** Rank Weighted Colocalization (RWC) coefficients for cis-NED 19 and Lysotracker fluorescent images.

	Lysosomes	NED-Positive Vesicles
RWC NED	0.703 ± 0.0005	0.63 ± 0.0006
RWC Lysotracker	0.707 ± 0.0005	0.63 ± 0.0005
